# A Repeated Lie Becomes a Truth? The Effect of Intentional Control and Training on Deception

**DOI:** 10.3389/fpsyg.2012.00488

**Published:** 2012-11-12

**Authors:** Xiaoqing Hu, Hao Chen, Genyue Fu

**Affiliations:** ^1^Department of Psychology, Zhejiang Normal UniversityJinhua, China; ^2^Department of Psychology, Northwestern UniversityEvanston, IL, USA

**Keywords:** training, intentional control, deception, instruction, differentiation of deception paradigm, automaticity

## Abstract

Deception has been demonstrated as a task that involves executive control such as conflict monitoring and response inhibition. In the present study, we investigated whether or not the controlled processes associated with deception could be trained to be more efficient. Forty-eight participants finished a reaction time-based differentiation of deception paradigm (DDP) task using self- and other-referential information on two occasions. After the first baseline DDP task, participants were randomly assigned to one of three groups: a control group in which participants finished the same task for a second time; an instruction group in which participants were instructed to speed up their deceptive responses in the second DDP; a training group in which participants received training in speeding up their deceptive responses, and then proceeded to the second DDP. Results showed that instruction alone significantly reduced the RTs associated with participants’ deceptive responses. However, the differences between deceptive and truthful responses were erased only in the training group. The result suggests that the performance associated with deception is malleable and could be voluntarily controlled with intention or training.

## Introduction

Despite the claim that there is no unique lie-specific characteristic associated with lying or deception, such as Pinochio’s nose (cf. Rosenfeld, 1995), it has been widely accepted that lying requires greater amount of cognitive control than telling the truth. Studies from developmental psychology found that children’s ability to tell lies are closely related with their development of executive control functions (Talwar and Lee, 2008). Studies from cognitive psychology similarly demonstrated that lying required more mental operations than truth (e.g., decisions to lie, construction of lying responses), which led to prolonged reaction times (RTs, e.g., Walczyk et al., 2003). Recently, research from cognitive neuroscience adds evidence that also supports the notion that lying is more task-demanding than truth: compared with truth, lying ubiquitously recruits brain regions that are involved in cognitive control such as the dorsolateral prefrontal cortex (DLPFC) and the anterior cingulate cortex (ACC, Spence et al., 2001; Langleben et al., 2002; Sip et al., 2008). A recent meta-analysis of neuroimaging of deception showed that the brain regions involved during lying are highly overlapped with the brain regions involved in executive functions, especially working memory and response inhibition (Christ et al., 2009).

This attribute of lying was recently utilized in applied research to aid in deception detection. For instance, it has been shown that people are generally not good at spotting liars via behavioral cues (Bond and DePaulo, 2006). However, it was found that people are more accurate in detecting lies when liars’ cognitive demand is high than when liars’ cognitive demand is low. This is based on the idea that as lying is already task-demanding, liars whose cognitive demand is particularly high would find it more difficult to manage lying as fewer resources are available, compared to liars whose cognitive demands are low, as relatively more resources can be used for lying (Vrij et al., 2008).

Although there are converging lines of evidence supporting the notion that lying is more task-demanding than truth-telling, this hypothesis should be investigated with more scrutiny given the recent evidences. Like many other complex social behaviors, lying is far from a uniformed homogenous behavior. There are increasing studies aiming to de-couple different sub-types of deceptions. For instance, people may tell lies either about others or about oneself; the event people may lie about could be experienced or not-experienced; the lies could also either be spontaneous or be well-practiced (Ganis et al., 2003, 2009; Abe et al., 2006; Johnson et al., 2008; Walczyk et al., 2009; Hu et al., 2011). These studies have consistently found that different types of lies showed different behavioral patterns, as well as non-overlapping neural activities. Specifically, lying about experienced events was associated with higher level of ACC activity compared to lying about not-experienced events, which is taken to suggest the former is associated with higher conflict (Abe et al., 2006). Moreover, it has been found that rehearsed, previously memorized lies were associated with less conflict compared with spontaneous lies, as evidenced by decreased activities in ACC (Ganis et al., 2003).

Although the abovementioned studies provided evidences that how cognitive demand may vary depending on different types of lies, whether or not the cognitive demand associated with lying can be intentionally reduced remains an open question. So far, only a few of studies investigated this issue. For instance, Johnson et al. (2005) found that although practice reduced the RTs of deceptive responses generally, the difference between deception and truth still remained. Walczyk et al. (2009) gave participants time to prepare and practice their lies before a cognitive lie detection test. Results showed that participants’ practiced deceptive responses were associated with reduced RTs than deceptive responses that had not been prepared nor practiced prior to the test (Walczyk et al., 2009, see also DePaulo et al., 2003, for preparation’s influence on liars’ behavioral cues). Another recent study manipulated the proportion of questions that required either honest or deceptive responses during a question set. It was found that when participants must deceive frequently in a question set, the lies became less task-demanding than when participants should tell the truth frequently. In other words, the more questions participants lied about, the easier it was to lie (Verschuere et al., 2011).

In the present study, we directly investigated whether or not lying can be trained to be more automatic and less task-demanding. We argued that since in most previous deception studies, participants were instructed to lie immediately after they receive the instruction, the lying can be classified as unpracticed. However, in real-life scenarios, liars may construct and practice lies before the interrogation. Indeed, practice or training may help people to improve the efficiency of knowledge retrieval, response inhibition and even working memory capacity across various task domains (Pirolli and Anderson, 1985; MacLeod and Dunbar, 1988; Milham et al., 2003; Olesen et al., 2004; Walczyk et al., 2009; Brehmer et al., 2012; Hu et al., 2012b). Since deception or lying has been conceptualized to rely on similar executive functions, especially working memory and response inhibition (Christ et al., 2009), it is possible that as these general-purposes processes (e.g., working memory, response inhibition) are malleable upon training, deception can also be trained to be more automatic. Thus, we hypothesized that participants who received training on deception would similarly find lying to be less demanding. Moreover, the post-training deception may not even be distinguished from truth.

In addition to the training condition, we also investigated the effect of instruction on deceptive responses. It has been found that giving participants specific instructions regarding response pattern can have considerable effects over participants’ behavioral performance (Verschuere et al., 2009; Hu et al., 2012b). Specifically, participants can significantly reduce their RTs in tasks involving response conflict and control upon mere instruction (Hu et al., 2012b). This instruction group is also necessary for us to examine whether or not behavior changes between pre- and post-test, if any, can be attributed to training or to experimental instructions. For instance, it has been argued that the benefits of training on participants’ performance may not necessarily due to the training itself, but can be due to factors such as participants’ expectations about improvements (e.g., Brehmer et al., 2012). Thus, the instruction manipulation allows us to investigate the effect of mere instruction over one’s deceptive responses. Furthermore, the comparison between the instruction group and the training group enables us to dissociate the behavioral change related to training from the change that is due to instructions. This may also provide us with a more detailed picture of the factors that may influence deceptions.

## Materials and Methods

### Participants

Forty-eight participants (nineteen males, average years = 22.23) from Jinhua, China were recruited via advertisements on campus and received monetary compensation for their time. They were randomly assigned to three groups (*N *= 16 in each group): control group (seven males, mean age = 22.13), instruction group (four males, mean age = 22.5), and training group (eight males, mean age = 22.25). Consent forms were obtained from participants before the experiment.

### Materials

Three pieces of personal information from each participant were collected for self-referential information list: full name, birth-date, and hometown. Next, a list of names, dates, and Chinese city/town names were provided to participants, who were instructed to select those with special personal meanings (e.g., a city may become relevant because participants’ relatives live there). Then three pieces of information, a name, a date, and a town name, were randomly selected from the list that contained only personal-irrelevant information. These three pieces of information were used as other-referential information.

Stimuli were presented as words using E-prime. Each item was presented for 15 times, resulting in a total of 90 trials [3(name, date, town) × 2(self-referential vs. other-referential) × 15] in one block. Stimulus was presented for 300 ms in white font against a black background on a computer monitor. The inter-stimulus-interval was randomly varied between 1500 and 2500 ms.

### Experimental procedural

The Differentiation of Deception Paradigm (DDP) was constructed following Furedy et al. (1988). The task consisted of two blocks: in the truthful block, participants were asked to respond to all stimuli honestly. They were asked to press one key indicating “self” to their self-referential information; and to press another key indicating “other” to the other-referential information. In the deceptive block, participants were asked to press “self” to the other-referential information and to press “other” to their own information, i.e., to pretend they were someone else while concealing their true identity. The order of the two blocks was counterbalanced across participants. Since participants may develop a response-mapping strategy from the first block to the second block by merely reversing the button press without experiencing being truthful or deceptive, another 30 trials of words “SELF” and “OTHER” were included in each block as catch trials. These catch trials were randomly interspersed among the self- and other-referential information. Specifically, participants were instructed to press the key indicating “self” to “SELF” catch trials and to press the key indicating “other” to “OTHER” catch trials. Importantly, this response-mapping was consistent across both truthful and dishonest blocks (for other types of catch trials, see Johnson et al., 2005; Hu et al., 2011). Thus, participants finished 120 trials (90 response trials and 30 catch trials) in each block. Speed and accuracy were equally emphasized.

Upon the completion of the baseline DDP for all participants, participants in the *control* group performed an irrelevant vision illusory task for 15 min, followed by a second DDP. In the irrelevant task, participants watched a series of apparent motion pictures and decided whether the dots on the picture were moving or not. The control group aimed to control for possible effect of task familiarity/fatigue over one’s behavioral performance across two tests.

For participants in the *instruction* group, their RTs and errors from the truthful and deceptive blocks of the baseline DDP they just finished were calculated and shown to them. Participants were debriefed regarding the meanings of these behavioral measures. They were explicitly told that their deception could be inferred from the increased RT and the decreased accuracy in the deceptive block compared to the RTs and accuracy from the honest block (in fact, every participants in the instruction group and the training group (described below) showed at least one of the two behavioral indicators associated with deception). Next they were instructed to try their best to speed up their RTs and to reduce possible incorrect responses during the deceptive block in the following DDP task. After the instruction was given, participants conducted the second DDP task in the same order as in the baseline DDP task.

For participants in the *training* group, everything was the same as in the instruction group, except that in addition to being instructed to speed up and be more accurate, they were given 360 trials (i.e., three deceptive blocks) that required deceptive response to improve their behavioral performance of the deceptive block. There were two intervals during the training session in which participants took a short break. After the training, participants proceeded to the second DDP in the same order as in the baseline DDP task.

## Results

**Figure 1:**
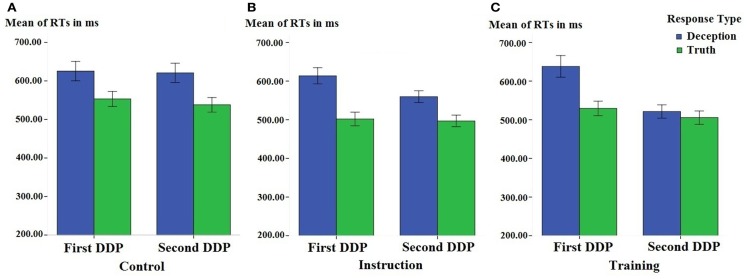
**Participants’ mean reaction times (RTs, in milliseconds) associated with deceptive and truthful responses in the first and the second differentiation of deception paradigm task, in the control (A), instruction (B), and training group (C), separately**. Error bars indicate ±1 Standard Error.

**Figure 2:**
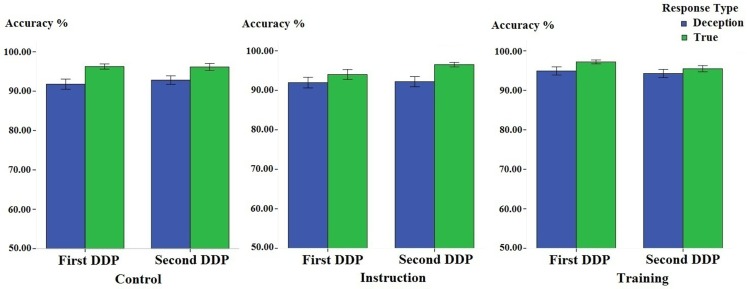
**Participants’ mean accuracy (in percentage) associated with deceptive and truthful responses in the first and the second differentiation of deception paradigm task, in the control, instruction, and training group, separately**. Error bars indicate ±1 Standard Error.

The behavioral data from the baseline DDP and the second DDP from three groups is presented in Figures 1 and 2. It can be observed that the baseline deception is associated with longer RT and reduced accuracy. However, the RTs of deception were reduced in both the training group and the instruction group.

To statistically test our hypothesis, we first conducted a mixed-model 3 (group as a between-subject variable: control vs. instruction vs. training) by 2 (response type as a within-subject variable: truth vs. deception) by 2 (time as a within-subject variable: first vs. second DDP) by 2 (stimulus type as a within-subject variable: self- vs. other-referential information) analysis of variance (ANOVA) on RTs of correct responses. This test yielded a significant main effect of response type [*F*(1, 45) = 116.19, *p *< 0.001, $\eta_{p}^{2}=0.72$], indicating that deception took significantly longer time than truth (Mean ± SE, 596.48 ± 11.76 vs. 520.79 ± 9.99 ms). The main effect of time was also significant, *F*(1, 45) = 29.06, *p *< 0.001, $\eta_{p}^{2}=0.39.$ This was due to participants’ faster RTs in the second DDP compared to the first DDP task (540.36 ± 9.96 in the second DDP vs. 576.92 ± 11.73 ms in the first DDP). Moreover, stimulus type was also significant, *F*(1, 45) = 10.14, *p *< 0.01, $\eta_{p}^{2}=0.18,$ as self-referential information had faster RTs than other-referential information (553.44 ± 10.19 vs. 563.84 ± 10.73 ms). Regarding interactions, a significant two-way, stimulus type by response interaction was significant [*F*(1, 45) = 83.56, *p *< 0.001, $\eta_{p}^{2}=0.65$]. This was because the RTs discrepancy between honest and deceptive responses for self-referential information was larger (497.96 ± 9.73 vs. 608.93 ± 12.19 ms) than for other-referential information (543.64 ± 10.62 vs. 584.04 ± 12.09 ms).

Most importantly, the three-way response × time × group interaction was significant, *F*(2, 45) = 8.26, *p *< 0.01, $\eta_{p}^{2}=0.27.$ No other effects were found significant. To understand this three-way interaction, we focused on the influence of time over response type by conducting 2 (first vs. second DDP) by 2 (deception vs. truthful responses) ANOVAs in three groups separately.

In the control group (see Figure 1A), there was no significant interaction between time and responses [*F*(1, 15) < 1, *p *> 0.5, $\eta_{p}^{2}<0.1$], suggesting that differences between deception and truth did not change across time.

In the instruction group (see Figure 1B), however, a significant time by response interaction was found: *F*(1, 15) = 12.16, *p *< 0.01, $\eta_{p}^{2}=0.45.$ This suggested that mere instruction would significantly influence participants’ behavioral performance of deception. To understand this interaction and to highlight our main variable of interest (i.e., differences between deceptive and honest responses), we calculated the differences between deception and truth blocks in the first and the second DDP separately. A paired sample *t*-test showed that participants who received the speed up instruction significantly reduced the differences between deceptive and honest responses from the first to the second DDP task (111.54 ± 12.98 vs. 62.73 ± 14.65 ms; *t*(15) = 3.49, *p *< 0.01, Cohen’ *d *= 0.88).

Even though instruction did reduce the differences between deceptive and truthful response from the first to the second DDP task, it remained to be investigated whether or not RTs can distinguish deceptive from honest response in the second DDP task. A paired sample *t*-test comparing RTs of deceptive and honest responses found that the RTs associated with deceptive response were still longer than the RTs associated with honest responses (559.65 ± 15.36 vs. 496.92 ± 14.89 ms, *t*(15) = 4.28, *p *< 0.001, Cohen’s *d *= 1.04). This pattern of results suggested that even though instruction did influence participants’ deceptive responses, it was not sufficient to eliminate the deception-truth differences.

In the training group (see Figure 1C), the same 2(time: first vs. second DDP task) × 2(response: deception vs. truth) within-subject repeated measure ANOVA resulted in a significant main effect of time: *F*(1, 15) = 26.33, *p* < 0.001, $\eta_{p}^{2}=0.64,$ suggesting that the training significantly reduced the RTs of the DDP task. The same test also revealed a significant main effect of response type [*F*(1, 15) = 20.02, *p* < 0.001, $\eta_{p}^{2}=0.57$], suggesting that deception and truth was significantly different. Most importantly, the time by response interaction was significant: *F*(1, 15) = 17.45, *p* < 0.001, $\eta_{p}^{2}=0.54.$

To understand this interaction, we calculated the RTs difference between deceptive and truthful responses (RT_deception_−RT_truth_) of the first and the second DDP tasks separately. It was found that this difference was significantly reduced after participants’ training from the first to the second DDP (108.67 ± 22.69 vs. 15.82 ± 10.93 ms, *t*(15) = 4.18, *p *< 0.001, Cohen’s *d *= 1.30). Moreover, in the second, post-training DDP, RTs for deceptive responses were not different from those of truthful responses (505.44 ± 17.39 for honest responses vs. 521.25 ± 17.13 ms for deceptive responses*, t*(15) = 1.45, *p *> 0.1, Cohen’s *d *= 0.23). In other words, training eliminated the difference between deceptive and truthful responses in the second DDP task.

Regarding accuracy (see Figure 2), the same condition by response by time by stimulus type mixed-model ANOVA revealed only a significant main effect of response type [*F*(1, 45) = 80.12, *p *< 0.001, $\eta_{p}^{2}=0.64$], indicating deceptive responses was less accurate than honest responses (0.93 ± 0.01 vs. 0.96 ± 0.01%). Neither other main effect nor interaction was significant (all *p*s > 0.05). The accuracy results suggested that there was no speed-accuracy trade-off.

## Discussion

The present data show that behavioral RTs performance associated with deception can be influenced significantly via instruction and training, as evidenced by significantly decreased RTs in the second DDP compared with the baseline performance. This pattern of results also shows that deception is not always associated with higher cognitive demand, as most previous studies suggested.

Results from the baseline DDP task replicated previous findings that lying usually produces prolonged RTs when compared with truth (Furedy et al., 1988; Walczyk et al., 2003; Hu et al., 2011; Verschuere et al., 2011). The prolonged RT and reduced accuracy are usually taken as indicators of high response conflict and cognitive control in tasks such as the Stroop task (MacLeod and Dunbar, 1988). Evidence from neuroimaing studies also demonstrated that when people generate deceptive responses in DDP tasks, the brain regions associated with cognitive control and conflict monitoring processes were more active than when participants give honest responses (e.g., Abe et al., 2006; for a meta-analysis, see Christ et al., 2009).

The present data, however, suggested that instruction and training could significantly decrease the task demand associated with deception as evidenced by reduced RTs. Specifically, the speed up instruction alone significantly reduced the difference between deceptive and honest responses. This result is also partially consistent with one recent study, in which it was found that instruction alone can result in speeding up RTs in an autobiographical implicit association test (aIAT) that involves response conflict and control (Hu et al., 2012b). Specifically, in an aIAT, participants are asked to perform a RT-based classification task that consists of four types of sentences: (1) true sentences (e.g., I *am in front of a laptop*), (2) false sentences (e.g., *I am climbing a mountain*), (3) crime-relevant sentences (e.g., *I stole a wallet*), and (4) crime-irrelevant sentences (e.g., *I read an article)*. It is hypothesized that for criminals, it will be easier to press the same button to both crime-relevant sentences and true sentences given that both have truth values (i.e., congruent responses) than to press the same button to both crime-relevant sentences and false sentences (i.e., incongruent responses that involve conflict). Thus, the aIAT examines the mental associations between criminal events and truth value. Hu et al. (2012b) found that participants who were instructed to speed up their RTs in the incongruent blocks were able to reverse the baseline results pattern, i.e., showing quicker responses in the incongruent blocks than congruent blocks, thus obtaining an innocent diagnosis.

However, in the present study, participants who were similarly instructed to speed up their responses in the deception blocks only reduced, but did not eliminate or reverse, the differences between deceptive and honest responses compared to the baseline results pattern. Given this discrepancy, it is possible that the influence of instruction over one’s performance depends on the nature of the specific type of response conflict and control involved in the task: in the autobiographical IAT task, the stimulus-response conflict involves in the incongruent responses concerns *recently established* mental associations (e.g., mock crime that was committed 10 min or weeks before the test, see also Hu and Rosenfeld, 2012); whereas in the self-other DDP task, however, the stimulus-response conflict involved in the deceptive responses concerns *long-term* mental associations (e.g., one’s self-referential information is always true). Indeed, De Houwer and colleagues found that participants could successfully fake their performance of an IAT that assessed one’s attitudes toward *novel* social groups (i.e., recently established mental associations, De Houwer et al., 2007).

Based on the discussion above, it is thus possible that the influence of instruction over performances in deception/response conflict tasks depends on the strength of mental associations: if the mental associations are newly acquired or recently established, it is likely that instruction alone will effectively help the participants to control the response conflict and behavioral performance. If the mental associations are established via long-term practice or socialization, however, it is likely that instruction itself is not sufficient for participants to overcome the response conflict involved in the task.

In addition to instruction, training here played an additive role in helping participants control their deceptive performance. Specifically, after participants were trained to speed up their responses in the deceptive block, the honest-deception differences in the baseline were eliminated in the post-training DDP task. As discussed above, controlling response conflicts that are generated from long-term associations (here self-referential information refers to “self” instead of “other”) may require training. Another example of response conflict that is generated from well-established association is the Stroop task (Stroop, 1935). In the Stroop task, people usually take longer to name the color of the incongruent word font (e.g., press the button indicating red color in response to the word “GREEN” printed in red color) than to name the word meanings since people (at least adults) are automatic in processing the meaning of the words (for a review, see MacLeod, 1991). Regarding whether or not training can reduce the Stroop effect, MacLeod and Dunbar (1988) employed a variant of the Stroop task and found that the stroop effect can be reversed only with extensive training as long as 20 h, rather than with relatively short training that last for 2 or 4 h.

Thus, though instruction alone is effective in reducing responses conflict that from recently established mental associations, training seems to be necessary in reducing conflicts that are from long established, well-practiced associations. Our results also extended another recent study, in which the deceptive responses could be made easier when the frequency of deceptive responses was increased in a question set (Verschuere et al., 2011). Together with these results, the current study, with an emphasis on training conducted within participants, supports the view that deception is malleable and its performance index can be voluntarily controlled to be more automatic.

One question arising here concerns the fact that deception seems to be more malleable than previous studies suggested (e.g., Johnson et al., 2005). Two possible reasons may be relevant: (1) previous studies showed that people may lie frequently in daily interaction (DePaulo and Bell, 1996). In other words, people may already “practice” lies in daily life, which makes lying more malleable; (2) more importantly, unlike previous studies in which participants merely repeated the tasks without an intention to improve (e.g. Johnson et al., 2005), participants in the present study practiced the deceptive responses with a conscious goal to speed up. Since mere practice (without an intention to improve) did not significantly change participants’ task performance (Hu et al., 2012b), it seems that instruction is a necessary element in training-induced behavioral change.

The present data may also shed light on deception detection studies. Specifically, if a deception detection study involves comparisons between unpracticed lying and truth-telling, then the results may not generalize to situations where well-practiced lies are involved (see also Walczyk et al., 2009). Thus, some preparations of lying may be profitably included in deception detection studies so as to increase ecological validity.

A related question is how to better detect prepared lies. Although we did not directly investigate this question here, some recent findings may be helpful: (1) as in Verschuere et al. (2011)’s study, adding filler questions that required honest responses may increase the lie-honest differences. This is based on the premise that increasing the predominance of one response mode (e.g., honest) should make the competing response mode more difficult (e.g., lies). Although participants may practice their lies before the test, some build-in filler questions that required honest responses during the test may make the prepared lies more difficult; similarly, Hu et al. (2012a) recently found that in a concealed information test, a higher number of irrelevant stimuli may make countermeasures or deliberate faking more difficult. (2) Although suspects can prepare some lies in anticipating certain questions, asking unanticipated questions for which liars may not be able to prepare may be helpful (see Vrij et al., 2009). A third strategy is to use certain algorithm to detect fakers based on their response patterns. For instance, Agosta et al. (2011) recently developed an algorithm to detect fakers in the aIAT contexts. Because of different task structures were used in the aIAT and the DDP, the algorithm cannot be directly applied here. Moreover, participants in the Agosta et al. (2011)’s study were asked to slow down their RTs to fake the test. A recent study showed that the algorithm based on slowing down RTs cannot be used in detecting fakers when they used the speeding up strategy, which was adopted in the present study (Hu et al., 2012b). Notwithstanding, future research should directly investigate whether or not prepared liars can be detected using certain abovementioned strategies.

A possible limitation of the present study is the relatively small sample size (*N *= 16 in each group) we used here. However, it should be noted that as the effect sizes we obtained here were large (given the effect is considered as large when Cohen’s *d *> = 0.8), and because large sample is usually required to observe small effect, we reasoned that the present sample size would not render our results unstable (see also Hu et al., 2012b). Nevertheless, future studies using large sample are necessary to replicate the effect we obtained here. Another possible caveat is the demand and expectancy effect may play a role here. However, it should be mentioned that unlike many psychological research in which the rationale/hypothesis of the study is concealed from participants, researchers in deception detection are usually interested in examining the extent to which participants can intentionally control their behavior during the test. This required participants to understand the rationale of the tests. This procedure is similar to many previous studies in deception detection studies involving countermeasures or deliberate faking strategies (e.g., Rosenfeld et al., 2004; Verschuere et al., 2009; Agosta et al., 2011; Hu et al., 2012b). Finally, it should be noted that although we obtained the instruction/training effect regarding RTs, we failed to find the similar pattern with accuracy. This may be due to the ceiling effect for accuracy results: in each group/condition, the accuracy was around 95%.

To conclude, this study showed that the performance of deception is malleable and becomes more automatic upon training. Meanwhile, instruction itself plays a significant role in inducing behavioral changes associated with deception. The results imply that future deception detection studies should take this variation of deception into account to better understand the complexity of lying and the corresponding behavioral/neural patterns, and to better identify liars.

## Conflict of Interest Statement

The authors declare that the research was conducted in the absence of any commercial or financial relationships that could be construed as a potential conflict of interest.
